# EXAMINING THE RELATIONSHIP BETWEEN PERSONALITY FACTORS AND NEUROCOGNITIVE FUNCTIONING IN DEMENTIA CAREGIVERS

**DOI:** 10.1093/geroni/igae098.2975

**Published:** 2024-12-31

**Authors:** Alayna Jump, Lauren Elliott, Sydnie Schneider, Elisabeth McLean, Lauren Chrzanowski, Amir Abu-Samaha, Volker Neugebauer, Jonathan Singer

**Affiliations:** Texas Tech University, Lubbock, Texas, United States; Texas Tech University, Lubbock, Texas, United States; Texas Tech University, Lubbock, Texas, United States; Texas Tech University, Lubbock, Texas, United States; Texas Tech University, Lubbock, Texas, United States; Texas Tech University, Lubbock, Texas, United States; Texas Tech University Health Sciences Center, Lubbock, Texas, United States; Texas Tech University, Lubbock, Texas, United States

## Abstract

More than 5.8 million Americans currently live with a neurodegenerative disorder such as Alzheimer’s Disease or Alzheimer’s Disease Related Dementia (AD/ADRD). Family caregivers of persons with AD/ADRD may be at increased risk for accelerated age-related cognitive decline compared to their peers. However, the risk and protective factors associated with neurocognitive functioning within this population are poorly understood. One factor yet to be explored is the relationship between personality traits and neurocognitive functioning. Certain personality traits, such as high neuroticism and lower openness have been linked to worse neurocognitive functioning in older adult populations, but these findings have not been expanded to family caregivers of persons with AD/ADRD. This study enrolled 52 family caregivers of persons with AD/ADRD who completed the NEO Five-Factor Inventory-3 (NEO-FFI-3), a measure of personality traits, and the Repeatable Battery for the Assessment of Neuropsychological Status (RBANS), a measure of overall neurocognitive functioning. A Pearson correlation revealed a positive trend (r(50) =.27, p =.054), such that individuals scoring lower on openness had worse neurocognitive functioning. However, the other four personality traits (i.e., neuroticism, agreeableness, extraversion, conscientiousness) were not significantly related to neurocognitive functioning (p’s >.27). Future studies should aim to investigate the relationship between personality factors and neurocognitive functioning within other samples as personality traits, which remain relatively stable over time, may provide medical providers the ability to easily identify individuals who might be more susceptible to neurocognitive decline, thus streamlining preventative measures.

